# Can Radiotherapy Be Omitted in Children With Hodgkin Lymphoma Who Achieve Metabolic Remission on Interim Positron Emission Tomography? Experience of a Tertiary Care Cancer Referral Center

**DOI:** 10.1200/JGO.2017.009340

**Published:** 2017-08-04

**Authors:** Sidharth Totadri, Venkatraman Radhakrishnan, Trivadi S. Ganesan, Prasanth Ganesan, Krishnarathnam Kannan, Kadur Mallaiah Lakshmipathy, Ganesarajah Selvaluxmy, Tenali Gnana Sagar

**Affiliations:** **All authors:** Cancer Institute (WIA) Adyar, Chennai, India

## Abstract

**Purpose:**

Treating pediatric Hodgkin lymphoma (HL) involves a delicate balance between cure and reducing late toxicity. Fluorodeoxyglucose positron emission tomography (PET) combined with computed tomography (CT) identifies patients with early response to chemotherapy, for whom radiotherapy may be avoided. The role of PET-CT in upfront risk stratification and response–adapted treatment is evaluated in this study.

**Methods:**

Patients with HL, who were younger than 18 years, were included. PET-CT was performed at baseline and after two cycles of chemotherapy. Patients were stratified into three risk groups: group 1 (stage I or II with no unfavorable features); group 2 (stage I or II with bulky disease/B symptoms); and group 3 (stage III/IV). A doxorubicin, bleomycin, vinblastine, dacarbazine–based regimen was used in early disease. A cyclophosphamide, vincristine, prednisolone, procarbazine, doxorubicin, bleomycin, vinblastine–based regimen was used in advanced disease.

**Results:**

Forty-nine patients were included. Fifteen (31%), seven (14%), and 27 (55%) patients were included in groups 1, 2, and 3, respectively. Among 36 patients who underwent staging by PET-CT at diagnosis, seven (19%) patients were upstaged and one (3%) patient was downstaged by PET compared with CT. On the basis of negative interim PET responses, 39 (80%) patients were treated without radiotherapy. The 3-year event-free survival for the entire cohort was 91% (± 5.2%) and overall survival was 100%.

**Conclusion:**

PET-CT is an excellent stand-alone staging modality in HL. The omission of radiotherapy can be considered in patients who achieve metabolic remission on interim PET.

## INTRODUCTION

Pediatric Hodgkin lymphoma (HL) is a highly curable malignancy. Survival rates exceed 90% with current treatment protocols.^1^ However, survivors continue to be at risk for therapy-related adverse events. Secondary malignancies, cardiovascular disease, and endocrinopathies contribute to late morbidity and mortality.^2,3^ Radiotherapy (RT) increases the risk of such events.^2^ Fluorodeoxyglucose (FDG) positron emission tomography (PET) combined with computed tomography (CT) is a robust staging modality for HL and enables risk-adapted therapy.^4^ In addition, it identifies patients with rapid, early response to chemotherapy and permits response-adapted therapy.^1,5^ PET-CT thus identifies patients who can potentially be treated without RT. This study evaluated the role of PET-CT in staging and response-adapted therapy in children with HL.

## METHODS

A retrospective file review was performed of consecutive patients younger than 18 years of age, diagnosed with classic HL who had completed therapy between January 2012 and December 2015. Patients with relapsed HL and those for whom PET-CT was not performed were excluded from the study. Informed consent was obtained from all patients. Diagnosis was by histopathology and immunohistochemistry performed on nodal biopsy. Baseline disease staging was performed using whole-body PET-CT and bone marrow trephine biopsy. The Cotswold modification of Ann Arbor staging was followed.^6^ Patients were stratified into three risk groups: group 1 (early-stage favorable), stage I or II, nonbulky, no B symptoms; group 2 (early-stage unfavorable), stage I or II, bulky and/or B symptoms; and group 3 (advanced stage), all stages III and IV. Bulky mediastinal disease was defined as a mediastinal mass with a horizontal tumor diameter more than one-third the thoracic diameter (measured transversely at the level of the dome of the diaphragm on an upright posterior-anterior chest x-ray). Bulky disease outside the mediastinum was defined as a single node or continuous nodal aggregate that measured > 6 cm in the longest diameter in any nodal area. Focal or multifocal FDG uptake in bone marrow was considered disease involvement. Diffusely increased bone marrow FDG uptake was not considered positive. Bone involvement was defined as FDG uptake correlated by tumor-typical correlation on CT. Four cycles of doxorubicin, bleomycin, vinblastine, dacarbazine (ABVD) chemotherapy were administered to patients in groups 1 and 2.^7,8^ Six cycles of cyclophosphamide, vincristine, prednisolone, procarbazine, doxorubicin, bleomycin, vinblastine (COPP-ABV) hybrid chemotherapy were administered to patients in group 3.^7,9^

**Fig 1:**
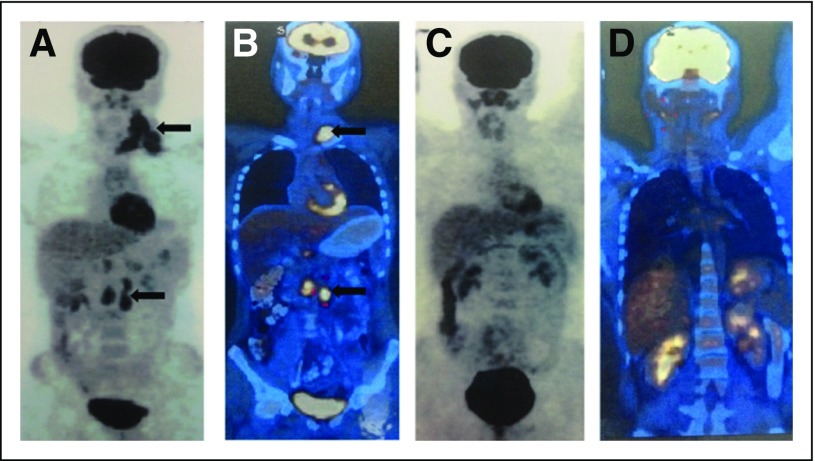
Interim positron emission tomography (PET) showing complete response compared with baseline PET. (A) Attenuated PET scan and (B) fusion PET–computed tomography scan at baseline showing stage III disease (sites of disease highlighted by black arrows). (C) Attenuated PET scan and (D) fusion PET–computed tomography scan obtained after two cycles of chemotherapy showing complete remission of disease.

Interim PET-CT was performed in all patients after the first two cycles of chemotherapy. Figure 1 shows baseline and interim PET scans in a patient with stage III disease. Baseline and interim PET-CT scans were evaluated and reported by a dedicated nuclear medicine unit. The PET-CT was scheduled to be performed 1 day before the 3rd cycle of chemotherapy and 2 weeks after exposure to the last chemotherapy. The Deauville five-point score was used for grading interim PET.^10^ In groups 1 and 2, a PET score of 1 to 2 was considered negative and a PET score of 3 to 5 was considered positive or suggestive of residual disease.^4^ In group 3, a PET score of 1 to 3 was considered negative and a PET score of 4 to 5 was considered positive.^4^ Because group 3 was already receiving 6 cycles of COPP-ABV, a higher threshold for escalation was used compared with groups 1 and 2. This approach, which limits toxicity in patients with advanced disease, has successfully been used in adults with HL in the same institute.^11^ In patients who were included in group 1, involved-field radiotherapy (IFRT) was restricted to those with positive interim PET response. In groups 2 and 3, IFRT was administered after completion of chemotherapy to patients with bulky disease at presentation as well as those with positive interim PET responses. RT was omitted for certain patients with upfront bulky disease and complete response on interim PET, per the discretion of the treating physician. Patients who initially had bulky disease at one site received IFRT at a dose of 20 to 30 Gy to the site that was bulky. Patients with residual disease on interim PET received IFRT at a dose of 30 to 36 Gy to the residual site(s) that showed FDG uptake on interim PET. Patients with metabolic progression on interim PET (in the form of new sites of disease) underwent treatment escalation to four cycles of escalated bleomycin, etoposide, doxorubicin, cyclophosphamide, vincristine, prednisolone, procarbazine (BEACOPP) regimen.^12^ In patients treated with escalated BEACOPP, end-of-treatment PET-CT was performed and RT was avoided if the Deauville score was 1.^11^

### Statistical Methods

For survival analysis, an event was defined as either death, relapse, or clinical progression. Overall survival (OS) was calculated from the date of diagnosis until the date of last follow-up. Event-free survival (EFS) was calculated from the date of diagnosis until the date of event. Baseline variables and outcomes were analyzed by descriptive statistics. Estimates of survival were computed using the Kaplan-Meier method. Statistical analysis was performed using SPSS software, version 17 (IBM, Chicago, IL).

## RESULTS

### Demographic Data

Forty-nine patients were diagnosed with HL during the study period. The mean age at diagnosis was 11.4 years (± 3.9 years; range, 4 to 17 years). The male to female ratio was 3.5:1. B symptoms were present in 21 (43%) patients. Bulky disease was identified in 19 (39%) patients. Seventeen (35%) patients had nodular sclerosis subtype and 31 (63%) had mixed cellularity subtype on histopathologic examination; one patient had HL not otherwise specified. Stage at presentation was I, II, II, and IV in five (10%), 17 (35%), 15 (31%), and 12 (24%) patients, respectively. Fifteen (31%), seven (14%), and 27 (55%) patients were included in risk group 1, 2, and 3, respectively. Table 1 summarizes the baseline and demographic data of patients.

**Table 1:**
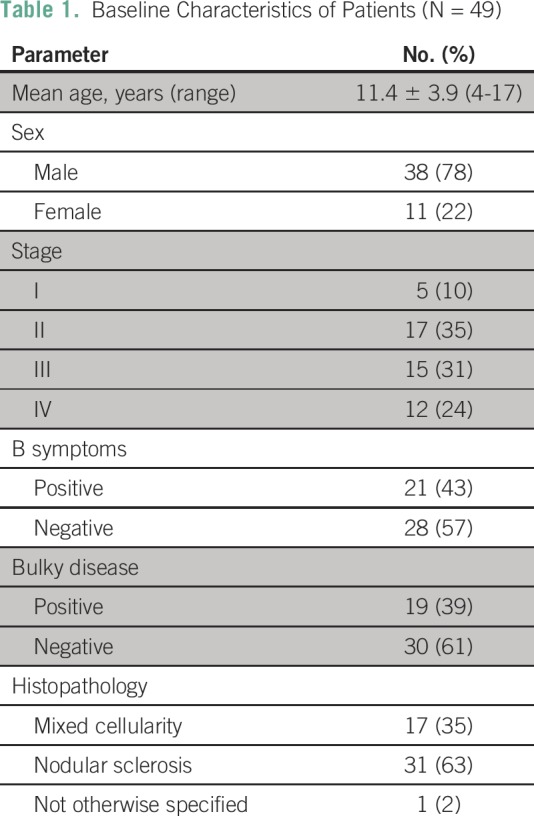
Baseline Characteristics of Patients (N = 49)

### Staging PET-CT

Of the 49 patients, 36 underwent upfront staging PET-CT. In the remaining 13 patients, staging was performed by contrast-enhanced CT because of resource limitations. Seven (19%) patients were upstaged by PET compared with CT. Of these seven patients, five were upstaged from stage III to stage IV; two patients were upstaged from stage II to stage IV. One (3%) patient was downstaged by PET from stage II to stage I. Consequently, the treatment group changed for two (6%) patients. New sites of disease identified by PET as compared with CT included bone marrow in 7 patients, spleen (5 patients), bone (1 patient), adrenal (1 patient), and parotid (1 patient). Three patients had lymphomatous infiltration in bone marrow on trephine biopsy. Focal marrow uptake on PET was observed in these three patients, in addition to four patients with negative trephine biopsy.

### Interim PET-CT in Groups 1 and 2

Interim PET-CT was performed in 48 patients. Thirteen patients who did not have PET-CT scans at baseline underwent PET-CT for interim assessment. As an individualized decision, one patient in group 1 was treated with 2 cycles of ABVD followed by IFRT with no interim assessment. All of the remaining 14 patients belonging to group 1 (100%) and 6 of 7 patients in group 2 (86%) had negative interim PET responses. RT was avoided in 14 (93%) patients in group 1 and in two (29%) patients in group 2 on the basis of negative interim PET responses. One of the two patients treated without RT in group 2 had bulky disease up front.

### Interim PET-CT in Group 3

**Fig 2:**
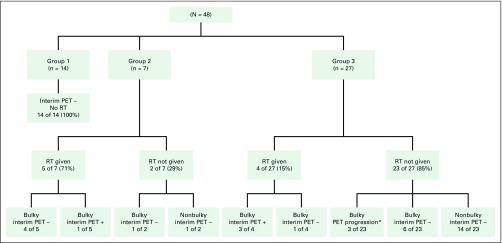
Flowchart depicting interim positron emission tomography (PET) response and administration of radiotherapy (RT) across the three risk groups on the basis of upfront bulky disease and/or interim PET response. Of the patients in groups 1, 2, and 3, 100%, 29%, and 85% were treated without RT, respectively. (*) Three patients in group 3 had evidence of progression on interim PET. These patients received 4 cycles of the bleomycin, etoposide, doxorubicin, cyclophosphamide, vincristine, prednisolone, and procarbazine chemotherapy regimen. Subsequent end-of-treatment PET responses were negative, and RT was avoided in these patients.

Three patients in group 3, who had metabolic progression on interim PET (new sites that were absent at baseline) went on to receive escalated BEACOPP. RT was not administered to them. Four patients received RT for upfront bulky disease. RT was avoided in 23 (85%) patients in group 3.Overall, 39 (80%) patients were treated without RT. Figure 2 illustrates interim responses and RT administration in the study participants. Ten of 19 (53%) patients with bulky disease were treated without RT.

### Survival Outcome

The mean and median durations of follow-up were 20.1 months (± 12.2 months) and 17 months, respectively. The 2-year OS and EFS rates in our study were 100% and 91% (± 5.2%), respectively. Two patients experienced disease relapse, and one patient had clinical progression. None of the patients who received escalated BEACOPP had clinical progression or relapse.

### Current Status of Patients Who Experienced Events

The patient included in group 2 who experienced progressive disease achieved partial response on interim PET. He subsequently, however, did not achieve remission but experienced disease progression despite salvage chemotherapy and administration of mantle-field RT. Because of poor results of autologous transplantation in the presence of gross residual disease, the patient is currently receiving metronomic chemotherapy with oral etoposide plus cyclophosphamide. The other two patients were included in group 3 and achieved partial responses on interim PET. PET-CT performed in the two patients after six cycles of COPP-ABV demonstrated complete remission. The patients experienced disease relapse 7 and 18 months, respectively, after initial diagnosis. One patient underwent salvage chemotherapy followed by autologous hematopoietic stem-cell transplantation and has had a disease free survival of 1 year. The other patient has received salvage chemotherapy and will subsequently undergo autologous hematopoietic stem-cell transplantation.

## DISCUSSION

A combined-modality approach in HL yields excellent response rates and EFS.^1^ Survivors of pediatric HL experience increased risk of secondary malignancies, cardiovascular dysfunction, and endocrinopathies throughout their lifetime.^1-3,13^ The tradeoff between cure and late toxicity necessitates the identification of patients who can be treated without RT and those who require intensification of chemotherapy and/or RT. This, in turn, requires a robust treatment approach on the basis of the upfront burden of disease (risk stratification) as well as early, favorable response to chemotherapy (response adaptation).^1,14^

The Lugano classification strongly recommends PET-CT for staging routinely FDG-avid histologic types, including HL.^4^ Several studies have established the superior sensitivity of PET-CT in the staging of HL compared with CT.^15-19^ PET-CT changes the stage in 10% to 30% of patients, with upstaging more common than downstaging.^4,5^ This does not significantly alter the management or outcome of disease.^4,5^ Our study reflected similar findings, with 19% of patients upstaged, 3% downstaged, and only two patients requiring allocation to a different treatment group. Extranodal sites of disease (including bone marrow, bone, and spleen), as well as unusual sites (such as parotid gland and adrenal gland), were identified by PET-CT. This reiterates the fact that PET-CT is superior to CT for recognizing extranodal disease.^20^ Trephine biopsy has conventionally been used in the staging of pediatric lymphomas. The procedure is often painful and traumatic. The superior sensitivity and negative predictive value of PET in diagnosing marrow infiltration obviates the need for this invasive test in patients with HL, per the Lugano classification.^4,21,22^ Therefore, PET-CT can be used as an accurate single-modality staging investigation for upfront staging and risk assessment in pediatric HL.

Treatment adaptation on the basis of anatomic response to chemotherapy has not yielded consistent results in pediatric HL. The Children’s Cancer Group trial CCG 5942 (A Randomized Comparison of Chemotherapy With and Without Radiotherapy for Children With Hodgkin’s Lymphoma: A Report from the Children’s Oncology Group) randomly assigned patients who achieved anatomic complete remission after receiving COPP/ABV hybrid chemotherapy to receive RT or no further therapy.^23^ The EFS was inferior without RT, although the OS did not differ significantly.^23^ In the prospective trial conducted by the German Pediatric Oncology Hematology Hodgkin Disease Study Group (GPOH-HD95; Treatment of Children and Adolescents With Hodgkin Lymphoma Without Radiotherapy for Patients in Complete Remission After Chemotherapy), RT was omitted for all patients who achieved complete remission after chemotherapy with vincristine, etoposide, prednisolone, doxorubicin, cyclophosphamide, and procarbazine.^24^ The progression-free survival (PFS) at 10 years was similar in patients with low-risk disease irrespective of omission of RT and significantly lower in patients with intermediate risk who did not receive RT. Although PFS was lower in patients with advanced disease who did not receive RT, the difference was not significant.^24^

Early interim PET performed after two cycles of chemotherapy has emerged as a strong and independent prognostic factor for predicting those patients who experience treatment failure and PFS.^4,5,25^ The translation of interim metabolic remission on PET to the elimination of RT from treatment needs to be further validated. The Children’s Oncology Group trial AHOD0031 (Dose-Intensive Response-Based Chemotherapy and Radiation Therapy for Children and Adolescents With Newly Diagnosed Intermediate-Risk Hodgkin Lymphoma) showed similar EFS with or without RT in patients whose PET showed rapid early response after chemotherapy with doxorubicin, bleomycin, vincristine, etoposide, prednisone, cyclophosphamide.^26^ The same trial also demonstrated improved survival by escalating chemotherapy in patients with PET-positive disease at the time of interim assessment.^26^ The St Jude Children’s Research Hospital–Stanford University–Dana-Farber Cancer Institute–consortium (Association Between Radiotherapy vs. No Radiotherapy Based on Early Response to VAMP Chemotherapy and Survival Among Children With Favorable-Risk Hodgkin Lymphoma) avoided RT in patients with low-risk HL who achieved complete remission with vinblastine, doxorubicin, methotrexate, and prednisone chemotherapy, with favorable results.^27^ The European Network for Pediatric Hodgkin Lymphoma conducted a large multicenter trial (Pediatric Hodgkin Lymphoma) on the basis of the vincristine, etoposide, prednisolone, doxorubicin, cyclophosphamide, dacarbazine chemotherapy backbone, in which RT was only administered to patients with positive interim PET responses.^1^ The results of this trial are awaited.

Two trials that were performed in adult HL—the Randomised Phase III Trial to Determine the Role of FDG–PET Imaging in Clinical Stages IA/IIA Hodgkin’s Disease (RAPID), and the European Organisation for Research and Treatment of Cancer/Lymphoma Study Association/*Fondazione Italiana* *Linfomi* H10 trial (Omitting Radiotherapy in Early Positron Emission Tomography–Negative Stage I/II Hodgkin Lymphoma Is Associated With an Increased Risk of Early Relapse)—showed a higher risk of progression of close to 5% on omission of RT in patients who achieved negative FDG-PET responses after two to three cycles of ABVD.^28,29^ The burden of late effects is much higher in children, because they have many more years of survivorship compared with adults. Extrapolation of results of adult trials to pediatric HL cannot be done blindly. ABVD and COPP-ABV continue to be used as common chemotherapy regimens in pediatric HL.^7,30^ The question of whether one can use interim PET response to eliminate RT in children treated with these regimens can only be answered by pediatric trials conducted on the basis of the same chemotherapy regimens.

The elimination of RT or treatment modification on the basis of interim PET response is yet to be validated as a standard of care. In our study, we were able to successfully treat 80% of our patients without RT, across all risk groups, on the basis of interim PET response. In patients with low-risk disease (group 1) all but one patient were treated without RT. In addition, 85% of patients with advanced disease were treated without RT. It is common practice to radiate sites of disease that are bulky up front. Jain et al^30^ administered RT to the majority of patients (71%) with upfront bulky disease, among 167 patients with HL who were treated with ABVD chemotherapy. Our treatment guidelines recommend RT in all patients with bulky disease at presentation.^7^ Following the availability of PET for interim response assessment, we considered the elimination of RT in patients with excellent metabolic response on interim PET, on a case-by-case basis. Despite treating a large proportion of our patients without RT, the EFS and OS were satisfactory. None of the patients with bulky disease and complete response on interim PET, who were treated without RT, experienced disease relapse during the study period. The limitations of our study include the small sample size and short duration of follow-up. In the era before the availability of PET for staging and response assessment, studies reported successful management of pediatric HL without or with restricted use of RT.^9,31^ However, six to eight cycles of chemotherapy regimens such as ABVD and COPP-ABV were administered, potentially predisposing the survivors to late effects including anthracycline-induced cardiotoxicity and alkylating agent–induced gonadal toxicity.^9,31^

Most international trials in children, which are currently evaluating the role of interim PET in treatment adaptation, do not use ABVD chemotherapy.^1,26,27^ However, the ABVD regimen is still used in several centers. In developing countries such as India, the regimen is popular because of reduced acute toxicity, ease of administration, ready availability, and effectiveness.^30^ It is difficult to extrapolate the results of trials evaluating interim PET–based treatment adaptation, using different chemotherapy regimens, to those with patients receiving ABVD. Our study demonstrates the feasibility of taking such an approach in patients being treated with ABVD. Eliminating RT during the treatment of childhood HL is a significant step toward reducing late toxicity. A prospective, randomized, multicenter trial is required to assess the role of interim PET–based treatment modification and elimination of RT in children with HL receiving ABVD chemotherapy.
